# Discrepancy Between Chargemaster Prices and Hospital Quality for Cataract Surgery

**DOI:** 10.7759/cureus.74217

**Published:** 2024-11-22

**Authors:** Avery Morrison, Abha Amin, Steve Park, Joseph Branco, Richard S Chaudhary, Jai Ahluwalia, Cara L Grimes

**Affiliations:** 1 Department of Ophthalmology, New York Medical College, Valhalla, USA; 2 Department of Ophthalmology, Westchester Medical Center, Valhalla, USA; 3 Department of Medicine, Beth Israel Deaconess Medical Center, Harvard Medical School, Boston, USA; 4 Department of Obstetrics and Gynecology and Urology, New York Medical College, Valhalla, USA

**Keywords:** cataract surgery, chargemasters, healthcare economics, healthcare quality, price transparency

## Abstract

Background

The United States continues to rank as one of the most expensive healthcare systems in the world, and cataract surgery, the most commonly performed surgery, is one of the primary drivers of healthcare expenditure. Increasing efforts have been made to try to minimize U.S. healthcare spending, such as the 2018 Executive Order requiring hospitals to publish a machine-readable list, a *chargemaster*, of prices for all offered procedures to increase price transparency and reduce healthcare spending. Given cataract surgery is highly standardized with predictable costs, the goal of this study was to analyze pricing variability for cataract surgery across the United States and determine if there is a relationship between listed chargemaster prices and hospital characteristics or quality.

Methodology

In this cross-sectional study, all available chargemasters were downloaded in the Spring of 2019 for hospitals across California, Massachusetts, Mississippi, New York, and Ohio, which were selected to represent different regions of American healthcare. An electronic search algorithm was developed to search each chargemaster using Current Procedural Terminology (CPT) codes and specific terms to extract pricing data, such as CPT codes: 66984 and 66982, and search terms: “xcapsular”, “cataract”, “extracapsular”, “xtracaps”, “phacoemulsification”, “lens extraction”. Listed hospital characteristics were also collected, such as hospital type, ownership, and Centers for Medicare & Medicaid Services (CMS) star rating; urban or rural location; critical access status; and whether the hospital was involved in the training of residents enrolled in an approved graduate medical education program.

Results

All chargemasters (*n* = 825) were available and downloadable from hospitals in the selected states. Price listings for cataract procedures differed significantly across the five states included in our analysis (*P *< 0.001). Price listings were highest for California hospitals ($3,240.0) and lowest for Ohio hospitals ($1,268.6), which represented an 87% difference in median prices listed. When price listings were stratified by the CMS Star Quality Rating of the hospital, no significant linear differences were found, and interestingly, the highest quality hospitals (CMS star ranking of 5) had the lowest median of the mean price listed ($1,938.0). General acute care hospitals had the highest median of the mean price listed ($2,370.1) and hospitals run by the state and local government had the highest median of the mean prices listed, $3,254.8 and $4,059.4, respectively.

Conclusions

Cataract surgery prices varied significantly across the five states chosen to reflect the diversity of the U.S. healthcare system and hospitals, with the highest CMS Quality Star Rating hospitals having the lowest listed cataract surgery prices. This study highlights the disconnect between cost and quality of care, justifying the need for further investigation into what factors truly underlie hospital chargemaster prices. It presents an opportunity to reduce healthcare spending, without fear of losing quality of care.

## Introduction

The United States remains one of the most expensive healthcare systems globally. In 2021, the United States spent 17.8% of gross domestic product (GDP) on healthcare, nearly twice as much as the average Organization for Economic Cooperative and Development (OECD) country [1]. Tremendous efforts have been made to minimize how much the United States spends on healthcare. In 2018, the Trump Administration published an executive order focused on increasing price transparency to lower prices through improved consumer awareness of procedure costs and marketplace fair competition for services. This order required hospitals to publish a machine-readable list, a *chargemaster*, of prices for all offered procedures by January 2019 [2,3].

Chargemasters, however, have proven to be difficult to navigate and often do not offer true pricing transparency. Each hospital’s chargemaster is unique, with each having its own format of listed procedures and prices, which can be difficult to understand even for trained researchers. In addition, chargemasters often do not reflect the final amount a consumer will pay for a procedure. Prices listed could be vastly different from what consumers are billed because the prices listed are a starting point for reimbursement negotiations between hospitals and insurance companies [4]. Although the prices listed on each chargemaster may not represent the final bill, the listed prices have been shown to directly correlate with higher costs paid by consumers and insurance companies [2].

Cataract surgery is one of the primary drivers of healthcare expenditure because it is the most common surgery performed in the United States, with over 2 million cases performed each year [5]. This procedure is highly standardized and should have predictable costs; therefore, listed prices in each chargemaster should vary in pricing largely due to geographic factors [6]. To test this hypothesis, we analyzed listed chargemaster prices for cataract surgery across hospitals from five different states, which represent the diversity of the healthcare landscape. In addition, we gathered data on each hospital’s characteristics to determine whether price listings for cataract surgery have any relationship with hospital characteristics or quality.

## Materials and methods

In this cross-sectional study, differences in the price of cataract surgery as listed on hospital chargemasters were assessed in relation to several different factors. These factors included hospital type, ownership, and Centers for Medicare & Medicaid Services (CMS) star rating; urban or rural location; critical access status; and whether the hospital was involved in the training of residents enrolled in an approved graduate medical education program. We also evaluated geographical differences in chargemaster price listings for cataract surgery by obtaining price data for hospitals in the five selected states of California, Massachusetts, Mississippi, New York, and Ohio to broadly represent different regions of American healthcare. This research was declared exempt from Institutional Review Board (IRB) approval by the New York Medical College IRB since it did not meet the definition of research involving human subjects as defined by federal regulation 45 CFR Part 46 (IRB #12942). 

Selection of states

To represent the four different quartiles of health system performance as categorized by the 2019 Scorecard on Health System, we selected at least one state from each quartile (Table 1) [7]. 

**Table 1:** State selection based on quartile ranking from the Scorecard on Health System Performance by the Commonwealth Fund.

State	California	Massachusetts	Mississippi	New York	Ohio
Region	West	New England	Southeast	Mid-Atlantic	Great Lakes
Quartile	2nd Quartile	1st Quartile	4th Quartile	2nd Quartile	3rd Quartile
National Rank	14 (tied)	2	51	14 (tied)	33

Hospital selection

A list of all hospitals located in California, Massachusetts, Mississippi, New York, and Ohio was generated using data available on the Homeland Infrastructure Foundation website [8]. Closed, pediatric, psychiatric, and specialty hospitals, and long-term care, rehabilitation, and chronic care facilities were not included in this list since cataract surgery is either not or very rarely performed at these institutions. 

Downloading chargemasters 

Chargemasters were downloaded in the spring of 2019, reformatted to standard specifications, and stored before statistical analysis. Most chargemasters were downloaded by directly accessing individual hospitals’ websites, but for the state of California only, many chargemasters were taken from a centralized website managed by the Office of Statewide Health Planning and Development (OSHPD) [9]. Several hospitals included in our analysis were part of a larger healthcare group or network. Chargemasters were not available for individual hospitals in these networks, so the main chargemaster published by the network or group itself was used. 

Extracting price listings

An electronic search algorithm was developed to search each chargemaster using Current Procedural Terminology (CPT) codes and specific terms to extract pricing data. Price listings for cataract surgery were examined using CPT codes 66984 and 66982, along with search terms, including “xcapsular”, “cataract”, “extracapsular”, “xtracaps”, “phacoemulsification”, “lens extraction”.

Hospital characteristics

We used the Homeland Infrastructure Foundation GeoPlatform to determine the characteristics of all hospitals included in our study [10]. Characteristics we chose to include in our analysis were hospital size (determined by number of beds), ownership (government-owned, non-profit, or for-profit), type (general acute care, military, special, women’s, or critical access), presence of a helipad, and location in either an urban or rural environment. To further investigate the relationship between chargemaster prices and urban/rural location of hospitals, information was also obtained from the U.S. Department of Agriculture database published in 2013, which assigns hospitals a rating of 1-3 for urban counties and 4-9 for rural counties [11].

Hospital quality

We used CMS star ratings obtained from the CMS Hospital Compare Database to compare price differences according to hospital quality [12]. CMS Hospital Compare is a resource that compiles 57 different measures of hospital metrics and outcomes including mortality, readmission rates, patient experience, and patient satisfaction to calculate a star rating from 1 to 5, with 5 representing the highest quality of care. CMS star quality ratings are used to determine hospital reimbursement and have been found to effectively and legitimately reflect hospital quality [13].

Statistical analysis

For continuous variables, we initially calculated the minimum, maximum, and mean prices for cataract procedures within each subgroup for a particular variable for all hospitals included in that subgroup. We then individually calculated median values for the minimum, maximum, and mean prices we collected since price listings were nonparametric. Categorical variables were described using proportions. Comparisons were made using chi-square, one-way analysis of variance (ANOVA), or Kruskal-Wallis analyses.

## Results

Of the 1,000 hospitals selected from the five states used in our study, 825 chargemasters were publicly available. A total of 366 (44.4%) were from hospitals in California, 63 (7.6%) from Massachusetts, 80 (9.7%) from Mississippi, 172 (20.8%) from New York, and 144 (17.5%) from Ohio (Table 2). 

**Table 2:** Chargemaster availability and price listings ($) of hospitals by state. The data are represented as *n* and %. Comparisons were made using Kruskal-Wallis analyses. CA, California; MA, Massachusetts; MS, Mississippi; OH, Ohio; NY, New York

	CA	MA	MS	NY	OH	Notes
Number of chargemasters available	366 (44.4%)	63 (7.6%)	80 (9.7%)	172 (20.8%)	144 (17.5%)	*n *= 825
Cataract surgery price listed	3240.0	1882.5	2339.3	1937.0	1268.6	*P *< 0.001

Hospital characteristics, such as size, ownership, type, presence of helipad, and urban/rural location all differed significantly (*P *< 0.001; Table 3). New York had the largest hospitals, with an average number of 260 beds. Massachusetts had the highest percentage of non-profit hospitals (62, 77.5%), while California had the highest percentage of for-profit hospitals (99, 22.5%). Mississippi had the highest percentage of local-government-owned hospitals (41, 41.4%) and the most critical access hospitals (24, 24.2%). Massachusetts had the highest percentage (80, 100%) of hospitals with a helipad, whereas New York had the lowest (213, 84.9%). Regarding geographic location, Massachusetts (77, 96.3%) had the highest percentage of hospitals classified as urban, whereas most hospitals in Mississippi (72, 72%) were classified as rural.

**Table 3:** Hospital characteristics (significant at P < 0.001). The data are represented as *n* (number of hospitals) and %. Data are reported as median (range). Comparisons were made using Kruskal-Wallis analyses. CA, California; MA, Massachusetts; MS, Mississippi; OH, Ohio; NY, New York

Characteristics by state						
	CA	MA	MS	NY	OH	*P*-value
Hospital characteristics						
Average number of beds, *n*, median (range)	*n* = 434, 196 (169)	*n *= 75, 203 (169)	*n *= 96, 124 (151)	*n *= 198, 260 (226)	*n *= 179, 233 (235)	<0.001
Hospital ownership						
District Government (1)	48	3	1	5	4	<0.001
Federal Government (2)	18	5	3	13	6	
Local Government (3)	28	0	41	15	10	
State Government (4)	5	0	4	4	4	
Non-profit (5)	242	62	30	172	147	
For-profit/Proprietary (6)	99	10	20	0	13	
Hospital type						
General acute care (1)	434	75	72	176	144	<0.001
Critical access (2)	0	0	24	18	33	
Military (3)	18	5	2	13	6	
Special (4)	0	0	0	4	6	
Women (5)	1	0	1	2	0	
Miscellaneous						
With helipad, *n* (%)	453 (90.6)	80 (100)	100 (97.1)	213 (84.9)	189 (98.2)	0.003
Urban/Metropolitan (1)	418	77	28	171	129	<0.001
Rural/Non-metropolitan (2)	35	3	72	42	60	<0.001

Population characteristics, including average per capita income (*P *< 0.001), race (*P *< 0.001), percent uninsured (*P *< 0.001), and percent below the poverty level (*P *< 0.001) also all differed significantly (Table 4). Massachusetts had the highest ($71,831) listed income, and Mississippi had the lowest ($36,457). California had the highest percentage of individuals identifying as Hispanic or Latino (37.9%), Mississippi had the highest percentage of African American individuals (41.2%), and Ohio had the largest percentage of White individuals (83.7%). Regarding socioeconomic status, Massachusetts had the lowest percentage of insured individuals and individuals living below the poverty line, while Mississippi had the highest percentage of both insured individuals (15.1%) and people living below the poverty line (22.3%; Table 4). Overall hospital quality, as measured by the average overall CMS Quality Star Rating, was highest in Ohio (3.53) and lowest in New York (2.23).

**Table 4:** Population demographics (significant at P < 0.001). The data are represented as *n* (number of hospitals) and %. Data are reported as median (range). Comparisons were made using Kruskal-Wallis analyses. CA, California; MA, Massachusetts; MS, Mississippi; OH, Ohio; NY, New York

	CA	MA	MS	NY	OH	*P*-value
Population characteristics						
Number of hospitals (*n*)	320	69	60	166	121	
Average per-capita income ($)	62,336 (21,918)	71,831 (14,871)	36,457 (5,021)	64,361 (37,711)	46,577 (7,253)	<0.001
Total population	3,477,056 (3,858,985)	839,274 (466,600)	67,008 (69,252)	868,565 (817,766)	391,223 (457,238)	<0.001
Population characteristics (%)						
Hispanic or Latino	*n *= 453, 37.9 (14.6)	*n *= 80, 13.4 (7.95)	*n *= 100, 2.96 (2.17)	*n *= 213, 14.82 (12.75)	*n *= 189, 3.96 (2.40)	<0.001
White	*n *= 453, 73.60 (10.66)	*n *= 80, 79.59 (9.63)	*n *= 100, 55.95 (19.81)	*n *= 213, 77.19 (15.61)	*n *= 189, 83.72 (11.90)	<0.001
African American	*n *= 453, 6.00 (3.41)	*n *= 80, 10.12 (7.33)	*n *= 100, 41.21 (20.45)	*n *= 213, 13.31 (10.73)	*n *= 189, 11.65 (10.41)	<0.001
American Indian and Alaskan Native	*n *= 453, 1.88 (1.28)	*n *= 80, 0.53 (.26)	*n *= 100, 0.84 (2.51)	*n *= 213, 0.90 (0.88)	*n *= 189, 0.30 (.08)	<0.001
Asian	*n *= 453, 14.13 (9.51)	*n *= 80, 7.02 (4.26)	*n *= 100, 0.76 (0.70)	*n *= 213, 6.05 (6.03)	*n *= 189, 2.00 (1.69)	<0.001
Native Hawaiian or Pacific Islander	*n *= 453, 0.48 (0.28)	*n *= 80, 0.11 (0.05)	*n *= 100, 0.06 (0.06)	*n *= 213, 0.11 (0.09)	*n *= 189, 0.06 (0.06)	<0.001
Uninsured	*n *= 453, 8.31 (1.90)	*n *= 80, 3.42 (0.65)	*n *= 100, 15.08 (1.96)	*n *= 213, 5.69 (1.50)	*n *= 189, 7.81 (1.65)	<0.001
Poverty	*n *= 453, 13.15 (3.80)	*n *= 80, 10.74 (4.11)	*n *= 100, 22.32 (6.70)	*n *= 213, 13.36 (4.72)	*n *= 189, 14.13 (4.02)	<0.001
Hospital quality						
Average CMS quality star rating	*n *= 320, 2.99 (1.14)	*n *= 69, 3.07 (1.18)	*n *= 60, 2.68 (1.05)	*n *= 166, 2.23 (1.07)	*n *= 121, 3.53 (0.96)	<0.001

Price listings for cataract procedures differed significantly across the five states included in our analysis (*P *< 0.001) (Table 2). Price listings were highest for California hospitals ($3,240.0) and lowest for Ohio hospitals ($1,268.6). When price listings were stratified by the CMS star quality rating of the hospital, no significant linear differences were found (Table 5). Interestingly, the highest quality hospitals (CMS star ranking of 5) had the lowest median of the mean price listed ($1,938.0), while hospitals with a CMS Quality Rating of 3 had the highest median of the mean price listed ($2,477.2). There was no significant difference in price listings when considering urban/rural status as dichotomous (urban/rural) or as a continuum (Tables 6-7). General acute care hospitals had the highest median of the mean price listed ($2370.1) (Table 8). Finally, hospitals run by the state and local government had the highest median of the mean prices listed, $3,254.8 and $4,059.4 respectively (Table 9).

**Table 5:** Price listing of cataract surgery by CMS star rating ($). The data are represented as *n*. Comparisons were made using Kruskal-Wallis analyses. CMS, Centers for Medicare & Medicaid Services

	1	2	3	4	5	*P*-value
Cataract surgery	2079.3	2470.5	2477.2	2346.1	1938.8	0.718

**Table 6:** Price listing of cataract surgery by rural/urban status ($). The data are represented as *n*. Comparisons were made using Kruskal-Wallis analyses.

	Urban	Rural	*P*-value
Cataract surgery	2283.5	1805.2	0.338

**Table 7:** Price listing of cataract surgery by rural/urban continuum scale ($). Data are represented as N. Comparisons were made using Kruskal-Wallis analyses.

	1 (Most urban)	2	3	4	5	6	7	8	9 (Most rural)	*P*-value
Cataract surgery	2562.6	1644.8	3888.0	1398.0	2986.0	1398.2	1912.6	3951.1	.	0.866

**Table 8:** Price listing of cataract surgery by hospital type ($). The data are represented as *n*. Comparisons were made using Kruskal-Wallis analyses.

	General acute care	Critical care	Military	Special	Women	*P*-value
Cataract surgery	2370.1	1051.0	.	1900.0	.	0.351

**Table 9:** Price listing of cataract surgery by hospital ownership ($). The data are represented as *n*. Comparisons were made using Kruskal-Wallis analyses.

	District Government	Federal Government	Local Government	State Government	Non-profit	For-Profit (Proprietary)	*P*-value
Cataract surgery	2173.0	.	4059.4	3254.8	2086.6	1306.3	0.783

## Discussion

In summary, chargemaster cataract surgery prices varied significantly across the five states selected to reflect the U.S. healthcare system. We found that hospitals with the highest CMS quality star rating had the lowest listed cataract surgery prices. 

Our results indicate that there is significant variation in cataract surgery pricing across the United States. The highest listed price for cataract surgery across the five states was in California at $3,240 and was lowest for Ohio hospitals at $1,268, which represents an 87% difference in median prices listed. California’s higher prices can be partially explained by the high cost of living in California and the large percentage of urban hospitals (Table 3) [14,15]. 

However, other studies have found that there is a poor correlation between listed cataract prices and CMS’s Geographic Practice Cost Indices (GPCI) [16]. GPCIs are adjustments given to account for geographic differences for each cost relative to the national cost of each component. This poor correlation indicates that geographic variation alone may not explain the 87% difference in median prices listed between California and Ohio.

One of the most striking findings from this study is that the listed price did not directly correlate with the CMS quality star rating. For example, Ohio had the highest average CMS quality star rating across all five states, yet had the lowest median cataract surgery price listed, and hospitals with a CMS quality star rating of 5 had the lowest median price listed. This highlights the disconnect between cost and quality of healthcare. This reaffirms the need to further investigate the factors that influence healthcare pricing and presents an opportunity to lower healthcare spending without fear of jeopardizing the quality of care. Additionally, this implies that increased access to price transparency data empowers patients to make more informed economic care decisions and access higher-quality care. 

Prior studies corroborate our findings in the variability of chargemaster prices and CMS quality star ratings. A report published in 2021 found that cash price estimates varied 27-fold among hospitals for CPT code 66821 and 51-fold among hospitals for CPT code 66984. Further, this problem is not unique to ophthalmic procedures [17]. A 2020 report found that interhospital price variation ranged from 53% (transthoracic echocardiogram) to 243% (furosemide 40 mg) after analyzing chargemasters from the 500 top self-pay/uninsured revenue grossing hospitals nationally [18].

A 2021 study found that quality indicators did positively correlate with standard charges, such as mortality and readmissions, but negatively correlated with many others as we found in our study [16]. This difference can be explained by their analysis of a wide variety of procedures compared to our study on cataract surgery specifically.

Strengths and limitations

Little data exists analyzing publicly available chargemasters to investigate the price listing variations and discrepancies for cataract surgery across several states, hospital characteristics, and quality data. We present a comprehensive review of all available chargemasters from about 1,000 hospitals across states we believe accurately represent the healthcare diversity of this country. 

A limitation of this study is that the listed chargemaster pricing analyzed may not include other charges on a hospital bill for a given procedure, such as laboratory and supply fees, which could contribute significantly to the cost burden for a patient seeking cataract surgery. Further, listed chargemaster prices are often only a starting point for reimbursement negotiations and may not reflect the final amount paid by patients for a particular service or type of prosthetic lens, or even vastly differ. Aside from the financial complexity of chargemasters, patients face additional barriers while using chargemasters given their often knowledge of complex medical terms and CPT codes compared to trained research teams. This requires background knowledge to sift through chargemasters and find pricing information, making access to price transparency even more difficult. Lastly, correlation analysis concerning patient demographics and population characteristics was not conducted in this study.

Implications for future studies

Since the original mandate to publish chargemasters was released in 2018, a new mandate was issued by the White House on January 1, 2021, requiring hospitals to publish their insurance-negotiated rates. While this study gives us a framework that chargemasters poorly represent the quality or pricing consistency across states, further studies should investigate chargemaster pricing variation across listed insurance negotiated rates to give consumers a more accurate expectation of cost per procedure. Additionally, future studies should analyze additional quality measures and outcomes to understand their implications on the cost of care. This study represents a starting point in a focused effort to increase pricing transparency in cataract surgery. The methods used in this research can be applied to future chargemaster analyses.

## Conclusions

Cataract surgery prices varied significantly across the five states chosen to reflect the diversity of the United States healthcare system and hospitals with the highest CMS quality star rating had the lowest listed cataract surgery prices. This study accentuates the disconnect between cost and quality of care, justifying the need for further investigation. We need to understand what factors truly underlie hospital chargemaster prices and discover new opportunities to reduce healthcare costs and spending while compromising quality.
